# Relationships between Diffusion Tensor Imaging and Resting State Functional Connectivity in Patients with Schizophrenia and Healthy Controls: A Preliminary Study

**DOI:** 10.3390/brainsci12020156

**Published:** 2022-01-25

**Authors:** Matthew J. Hoptman, Umit Tural, Kelvin O. Lim, Daniel C. Javitt, Lauren E. Oberlin

**Affiliations:** 1 Clinical Research Division, Nathan S. Kline Institute for Psychiatric Research, Orangeburg, NY 10962, USA; umit.tural@nki.rfmh.org; 2 Department of Psychiatry, New York University Grossman School of Medicine, New York, NY 10016, USA; 3 Department of Psychiatry and Behavioral Sciences, University of Minnesota, Minneapolis, MN 55454, USA; kolim@umn.edu; 4 Schizophrenia Research Division, Nathan S. Kline Institute for Psychiatric Research, Orangeburg, NY 10962, USA; dan.javitt@nki.rfmh.org or; 5 Division of Experimental Therapeutics, College of Physicians and Surgeons, Columbia University, New York, NY 10032, USA; 6 Department of Psychiatry, Weill Cornell Medicine, New York, NY 10065, USA; leo4001@med.cornell.edu

**Keywords:** DTI, resting state, schizophrenia, FATCAT, tractography

## Abstract

Schizophrenia is widely seen as a disorder of dysconnectivity. Neuroimaging studies have examined both structural and functional connectivity in the disorder, but these modalities have rarely been integrated directly. We scanned 29 patients with schizophrenia and 25 healthy control subjects, and we acquired resting state fMRI and diffusion tensor imaging. We used the Functional and Tractographic Connectivity Analysis Toolbox (FATCAT) to estimate functional and structural connectivity of the default mode network. Correlations between modalities were investigated, and multimodal connectivity scores (MCS) were created using principal component analysis. Of the 28 possible region pairs, 9 showed consistent (>80%) tracts across participants. Correlations between modalities were found among those with schizophrenia for the prefrontal cortex, posterior cingulate, and lateral temporal lobes, with frontal and parietal regions, consistent with frontotemporoparietal network involvement in the disorder. In patients, MCS correlated with several aspects of the Positive and Negative Syndrome Scale, with higher multimodal connectivity associated with outward-directed (externalizing) behavior and lower multimodal connectivity related to psychosis per se. In this preliminary sample, we found FATCAT to be a useful toolbox to directly integrate and examine connectivity between imaging modalities. A consideration of conjoint structural and functional connectivity can provide important information about the network mechanisms of schizophrenia.

## 1. Introduction

Schizophrenia (SZ) is increasingly thought to be a disorder of brain dysconnectivity [1,2]. This idea is supported by MRI studies showing reduced white matter organization, using diffusion tensor imaging (DTI), and from other studies showing abnormal resting state functional connectivity (RSFC) in SZ compared to healthy controls. Many of these abnormalities are widespread throughout the brain, including between hemispheres, and are correlated with behavioral and psychophysiological deficits seen in the disorder, suggesting that they have clinical significance.

DTI studies have consistently observed abnormalities in FA associated with SZ [3,4,5,6]. These abnormalities have been related to visual [7,8] and auditory [9] processing deficits, poor cognitive task performance [10], and psychiatric symptoms [11,12,13]. Thus, white matter abnormalities are present in schizophrenia and have clinical relevance.

The DMN is a set of regions including the medial prefrontal cortex, the posterior cingulate/precuneus, left and right hippocampal regions, and the left and right inferior parietal regions [14,15]. This network shows heighted activity and functional connectivity during rest and during self-referential mental activity [15], and it is suppressed during cognitive challenges [16]. The DMN has shown abnormalities in SZ. Many studies have shown hyperconnectivity in SZ [17,18,19,20,21,22], and abnormalities in parts of the DMN are related to symptoms [20] and deficits [18] in the disorder. However, in some subgroups, lower connectivity has been found, for instance, among those with poor or moderate long-term clinical outcome [23]. Moreover, other studies have shown decreased RSFC in the DMN in schizophrenia [24,25,26]. Thus, although most studies show hyperconnectivity in the DMN in SZ, the precise nature of the abnormalities is unclear.

It has been suggested that RSFC might be related to underlying white matter connectivity [27]. Thus, regions that show RSFC might be connected by fiber tracts, such that the low frequency fluctuations are mediated by physical brain tracts. If they are uncorrelated, the RSFC might be driven by a third region, or the RSFC could be mediated by neurovascular coupling rather than structural connectivity. The reverse is also possible. In that case, there may be structural connectivity between regions that show RSFC in a frequency range that cannot be interrogated using BOLD fMRI.

The literature on direct relationships between structural and functional connectivity is limited. In 2009, Greicius et al. [27] demonstrated tracts between brain regions that are nodes of the DMN, suggesting coupling between functional and structural connectivity at a population level. Honey et al. [28] directly examined relationships between the two modalities and found that, in many cases, despite high RSFC, the measures were unrelated, with indirect connections playing an important role in explaining RSFC. Other studies, in both healthy samples and populations with medical illness, have used summary measures of structural-functional connectivity across the whole brain [29,30] or across neural networks (e.g., DMN, salience network (SN), and central executive networks (CEN), which comprise the triple network [31,32]), rather than within ROI pairs comprising a single network. 

Somewhat fewer studies have evaluated the correspondence between structural and RSFC in SZ. In a study comparing chronic and first episode patients with SZ, Kong et al. [33] found that coupling between RSFC and brain structure (in this case, gray matter volume) was higher in the former than the latter. Moreover, such coupling was lower in first episode patients than in healthy controls. Finally, coupling strength was positively correlated with Positive and Negative Syndrome Scale (PANSS) negative symptom scores. Another study estimated a global metric of whole-brain structural-functional coupling, as well as intra- and interhemispheric connectivity metrics, and estimates corresponded to fiber length (e.g., short, intermediate, and long fibers). Compared to healthy controls, offspring of parents diagnosed with SZ demonstrated increased structural-functional coupling in long-range fibers [34]. 

Other studies have also examined these issues in SZ [35,36,37,38]. Cabral et al. [36] found only weak correlations between structural and functional connectivities in SZ, whereas Nelson et al. [35] found evidence of reduced structural/functional coupling in SZ. In this study, implicated regions included insula, left middle temporal, right cuneus, and left lingual gyri, as well as fronto-striatal and fronto-temporal pathways. Cocchi et al. [37] found widespread relationships in healthy controls but reduced relationships in people with SZ in fronto-striatal, fronto-thalamic, and fronto-temporal networks. These studies used rather different methods, and some of the implicated systems differ across studies. The pattern of results across studies makes it clear that the relationship between structural and functional connectivity in SZ is nontrivial. Moreover, the nature and clinical significance of these relationships remain poorly understood. 

Recently, general-use programs have been developed to simultaneously estimate RSFC and tractography between elements of brain networks. Among these is the Functional and Tractographic Connectivity Toolbox (FATCAT; [39]). A few articles, using this method in psychiatric disorders, have been published (e.g., for prenatal alcohol exposure [40] and major depressive disorder [41]), but we are aware of none in SZ. Moreover, we are aware of no studies directly correlating structural and RSFC measures in the same region pairs within the DMN. Thus, although several large-scale brain networks show differences between healthy controls and people with SZ, we selected the DMN because it is robust and has several distinct parcels, which is critical for the assessment of structural and functional connectivity patterns. An understanding of the structural basis of DMN RSFC abnormalities in SZ might prove informative about the neural basis of the disorder.

Here, we apply FATCAT to RSFC and DTI data for homologous region pairs (i.e., the same pairs of regions) in the DMN in a proof-of-concept study of patients with SZ and healthy comparison subjects. We hypothesized that RSFC would be correlated with diffusion tensor parameters, differentially, between groups and that, in patients, structural-functional coupling would be related to psychiatric symptoms. 

## 2. Materials and Methods

### 2.1. Subjects

Participants were 33 patients with SZ, or schizoaffective disorder, and 31 healthy comparison participants. Of the 64 participants, 3 showed excessive motion artifact in the RSFC data, and 7 showed excessive artifact in both the DTI and RSFC data. The final sample was 29 patients and 25 healthy comparison subjects. Chronically ill patients were recruited from Rockland Psychiatric Center inpatient and outpatient units or had previously participated in studies at the Nathan Kline Institute (NKI). Diagnosis was confirmed by the SCID for DSM-IV-TR for Axis I disorder (Patient Edition (I/P) for patients and Non-patient Edition (I/NP) for healthy controls). There were 16 patients taking second generation antipsychotics. An additional 4 were on first-generation antipsychotic medications, and 9 were on a combination of the two. Healthy comparison subjects were recruited from the local community and had no presence or history of major Axis I DSM-IV-R diagnoses.

None of the participants had substance use disorders within the past 6 months, and current abstinence was verified by urine toxicology screen for outpatients and healthy comparison participants. Inpatients were presumed to be abstinent. Informed consent was obtained from all subjects involved in the study. The study was conducted according to the guidelines of the Declaration of Helsinki and approved by the Rockland Psychiatric Center/NKI Institutional Review Board. Resting state data (processed differently than herein) have been previously published [42,43,44,45,46,47,48,49]. 

Psychiatric symptoms were rated using the PANSS [50], which was available for 21 patients. Scores were derived for the White et al. [51] 5 Factor Model, which included Positive, Negative, Dysphoric Mood, Activation, and Autistic Preoccupation scores, with higher scores reflecting greater severity.

### 2.2. MRI Acquisition

MRIs were acquired at the Center for Biomedical Imaging and Neuromodulation at NKI using a 3T Siemens TiM Trio (Erlangen, Germany) and a 12-channel head coil. Resting state data were acquired using a 6-min echo-planar sequence (TR = 2000 ms, TE = 30 ms, matrix = 96 × 96, FOV = 240 mm, 34 2.8-mm slices, 0.7 mm gap, NEX = 180, GRAPPA = 2) with eyes closed. Wakefulness throughout the scan was verified by the MRI technologist. DTI was acquired using a twice-refocused spin echo sequence (TR = 9000 ms, TE = 84 ms, matrix = 128 × 128, FOV = 256 mm, 72 2-mm slices, 30 diffusion weighted images (*b* = 800 s/mm^2^), 7 images with *b* = 0 s/mm^2^, GRAPPA = 2; [52]). A T1-weighted anatomical image was acquired (MPRAGE; TR = 2500 ms, TE = 3.5 ms, TI = 1200 ms, matrix = 256 × 256, FOV = 256 mm, 192 1-mm slices). A field map was acquired to correct distortion in the resting state data (TR = 500 ms, TE = 4.92/7.38 ms, matrix = 96 × 96, FOV = 240 mm, 34 3.5-mm slice). 

### 2.3. Default Mode Network

**Figure 1:**
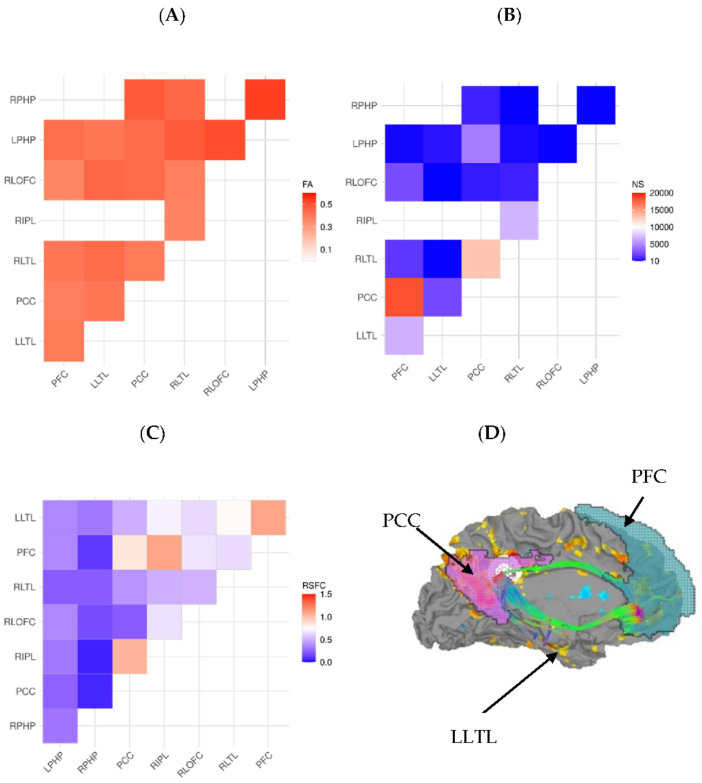
(**A**) FA for ROI-to-ROI tracts, (**B**) number of tracts for ROI-to-ROI pairings, (**C**) resting state functional connectivity connectomes for the default mode network of a representative participant. (**D**) Brain surface map, showing RSFC (yellow-blue color scheme) and superimposed tract (in green) for cingulate nodes of the DMN, projected onto the left medial surface of the brain in a representative subject. Labels for regions of interest are: LLTL = left lateral temporal lobe, PCC = posterior cingulate cortex, and PFC = prefrontal cortex.

The DMN was extracted from the Yeo 7-network “liberal” template [53], downloaded from https://surfer.nmr.mgh.harvard.edu/fswiki/CorticalParcellation_Yeo2011 (accessed on 23 November 2015). This template was registered using an affine-based nearest neighbor interpolation to the final resolution for all images. The network had 8 nodes (see inset Figure 1D for some of the nodes). These were isolated into separate regions using AFNI’s *3dClusterize* command individually for each participant. For tractography, these nodes were inflated up to the gray matter/white matter boundary using an FA threshold of 0.2.

### 2.4. DTI

DTI were processed using FATCAT’s routines. First, images were converted to NIFTI format. Then, slices and volumes with artifacts were automatically removed with *3dZipperZapper*, which identifies intravolume intensity variations and/or signal dropouts that are typical with high degrees of motion. This resulted in the exclusion of 2 participants (referred to in *Subjects* above) who retained fewer than 65% of volumes after this step. Data were eddy current and motion corrected and matched to a pseudo T2-weighted image that was created from the T1-weighted image that had been put into axial space by matching it to a T1-weighted standard image in ICBM 152 nonlinear symmetric 2009a atlas space (available at https://afni.nimh.nih.gov/pub/dist/doc/htmldoc/tutorials/fa%20tcat_prep/Prepreprocessing_I.html#example-setup (accessed on 18 March 2021)) using FATCAT routines and TORTOISE, v. 3.1.4 [54]. A weighting mask was used to address issues of low signal in subcortical regions (available from the same website). TORTOISE’s outputs were in 1.5 mm^3^ ICBM space. 

Susceptibility-based distortions were then corrected using Advanced Normalization Tools (ANTs) software [55]. In particular, the first *b* = 0 image was registered to the imitation T2 image, which had been resampled to the 1.5 mm^3^ resolution of the TORTOISE output. The transformation matrix was applied to the rest of the images in the DTI acquisition. Next, tensors were estimated using a nonlinear algorithm, along with eigenvalues, eigenvectors, and uncertainty distributions. Finally, tractography was computed using AFNI’s MINI protocol [56] with a resampling of 9 and an FA threshold of 0.2, using “AND” logic (i.e., tracts had to connect both ROIs) and a bundle size of ≥10 streamlines. For 9 of the 28 ROI pairs, at least 80% of subjects showed tracts meeting these criteria. Pairwise tracts were computed within the network. For each tract, FA and number of streamlines (NS) were estimated (see Tables S1 and S2 for between-group comparisons). From these, a connectome was generated for each participant and for each parameter. Statistical outliers (25–75%ile IQR ± 1.5 SD) were then removed for each variable. FA and NS were extracted and used in further analysis. 

### 2.5. RSFC

Resting state functional connectivity was examined using AFNI [57], FSL [58], and ANTs [55]. Prior to processing, the first five volumes were removed. Images were then motion-corrected, distortion-corrected using a field map, and smoothed with a 5 mm Gaussian kernel using FSL. We then used FSL’s ICA AROMA program [59] to automatically remove motion and other artifacts. In our data, the program generated between 19 and 48 components per subject, with 4–5 of them being retained. For interscan registration, the mean smoothed image was affine registered to the 1.5 mm^3^ resolution pseudo T2 image using ANTs, and the transformation matrix was applied to the denoised time series data, again using ANTs. The T1 image was segmented and used as regressors using AFNI’s *anaticor* algorithm [60]. White matter masks were eroded, and their time courses were extracted, along with CSF time courses, which were used as nuisance regressors. Six motion parameters from FSL were converted to AFNI’s format using code available at https://github.com/FCP-INDI/C-PAC/blob/master/CPAC/func_preproc/func_preproc.py#L237-L256 (accessed on 29 March 2021). Volumes with framewise displacement values of ≥ 0.5 were censored from the analyses, as was the preceding volume, with motion parameters and the white matter signal time course as additional nuisance covariates. Data were filtered at 0.01 to 0.1 Hz, and nuisance covariates were removed using 3dTproject.

From these images, the time series for each node of the DMN were extracted for use in RSFC computation using Pearson correlations. The resulting *r* values were converted to Z-scores to improve normality of distributions, which we denoted as RSFC(Z). From this, a DMN connectome was generated for each participant. Pairwise values were extracted for further analyses.

### 2.6. Multimodal Connectivity Scores (MCS)

For region pairs that showed significant correlations between DTI and resting state modalities in either group, we entered the data into principal component analyses, separately for patients and controls, as well as separately for FA and NS DTI variates. We termed the first component scores for each of these region pairs as multimodal connectivity scores (MCS).

### 2.7. Statistical Analyses

Distributions for each variable were checked for normality. The NS variables were all non-normally distributed. For these variables, we used Mann-Whitney U tests and Spearman correlations. In all other cases, we used Pearson correlations. To address the issue of missing data, regional values for RSFC(Z), FA, and NS were averaged across region pairs to generate an omnibus mean. Group differences for RSFC(Z) and FA were examined by t-test, whereas those for NS were tested using the Mann-Whitney U test. MCS variables were normally distributed. There were not tracts between every region pair for every subject, so the number varied across elements of the DMN connectome, and thus, sample sizes are indicated for each correlation. We used the Holm-Sidak false discovery rate (FDR) procedure [61,62] to correct for multiple comparisons. However, because of the preliminary nature of this study, we nonetheless present both FDR-corrected and uncorrected (*p* < 0.05) correlations to inform future studies.

To examine the effects of common covariates, for patients, we conducted Pearson (for FA) and Spearman (for NS) partial correlations with RSFC(Z) controlling for age, medication dosage (in CPZ equivalents), and intracranial volume (ICV; derived from Freesurfer). These are reported in Supplementary Tables S4 and S5 and are not corrected for multiple comparisons because they were exploratory analyses.

Parameter pairs that showed significant between-modality correlations, in participants with SZ or healthy controls, were examined for between-group differences in correlations. In addition, we examined the relationship between MCS and PANSS symptoms in correlation analysis. Tracts and RSFC(Z) were rendered in SUMA [63,64] for visualization, and heatmaps were prepared in R using the ggcorrplot package.

## 3. Results

### 3.1. Demographics

Demographic data for patients and controls are shown in Table 1.

**Figure 2:**
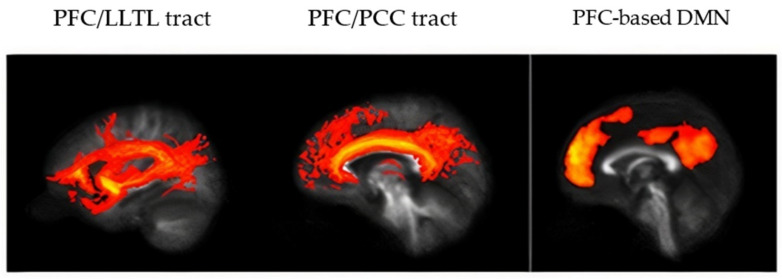
Representative data from a study participant. (**Left**) mean tract connecting PFC and LLTL for controls, (**Middle**) mean tract connecting PFC and PCC for controls, and (**Right**) Seed-based functional connectivity map of the DMN using the PFC ROI in controls, thresholded at RSFC(*Z*) = 0.4. Abbreviations are as in Figure 1.

Representative connectomes and images for modal participants are shown in Figure 1. Groupwise tracts and RSFC patterns are shown in Figure 2. Groupwise mean FA, NS, and RSFC(Z) are shown in Tables S1–S3. 

For RSFC(Z), the omnibus *t*-test was not significant (*t_51_* = −0.031, *p* = 0.98). However, RSFC(Z) was significantly higher for patients than controls in the prefrontal cortex/left lateral temporal lobe (PFC/LLTL) region pair (*t*_51_ = −2,69, *p* = 0.010, *d* = 0.74). 

For DTI measures, FA was significantly higher in controls than patients (*t*_51_ = 2.10, *p* = 0.041, *d* = 0.58). Specifically, FA was higher in controls than patients between the posterior cingulate cortex (PCC) and left parahippocampal gyrus (LPHP; *t*_51_ = 2.18, *p* = 0.034, *d* = 0.60) and between right lateral temporal lobe (RLTL) and right lateral orbitofrontal cortex (RLOFC; *t_50_* = 2.38, *p* = 0.021, *d* = 0.66). Similarly, NS was significantly higher in controls than patients (*U* = 234.0, *p* = 0.042, *r* = 0.28). In particular, NS was higher for controls than patients between the PFC and LLTL nodes (*U* = 181.0 *p* = 0.007, *r* = 0.38). 

### 3.2. DTI/RSFC(Z) Values and Correlations

Correlations between FA and RSFC(Z) for region pairs are shown in Table 2. For the right lateral temporal lobe/right inferior parietal lobule (RLTL/RIPL) pair, FA correlated negatively with RSFC(Z) in patients and this relationship differed significantly from controls (*t*_diff(50)_ = −2.10, *p* = 0.018). For controls, FA correlated positively with RSFC in the PCC/LPHP pair, which did not differ from that for patients (*t*_diff(50)_ = −1.47, *p* = 0.071). These correlations were not significant when corrected for FDR. The partial correlational analysis, depicted in Supplementary Table S4, did not reveal any significant results for homologous region pairs.

Spearman correlations between NS and RSFC for region pairs are presented in Table 3. Correlations for the PFC/PCC pair were significant in patients (*p*_FDR_ = 0.044, *p_uncorr_* = 0.005) and was significantly larger than in controls (t_diff(48)_ = −2.46, *p* = 0.002). Correlations between NS and RSFC(Z) were also significant for patients, but not controls, for the RLTL/RIPL (*p_FDR_* = 0.044, *p_uncorr_* = 0.006), the latter being the only significant negative correlation in patients. Both correlations in patients were significant after FDR correction. The correlation for the PFC/LLTL region pair differed between groups (t_diff(42)_ = −2.83, *p* = 0.002), with the correlation being negative in controls (*r* = −0.48, *p* = 0.022) and positive (but not significant) in patients. The Spearman partial correlational analysis depicted in Supplementary Table S5 showed similar results for homologous region pairs.

### 3.3. MCS

Principal component scores and eigenvalues are shown in Table S6. We examined relationships between MCS and clinical variables in correlational analyses among participants with SZ. Although these scores did not differ between groups, we examined relationships between them and psychiatric symptomatology in the patient group.

PANSS and MCS were uncorrelated for FA. PANSS Positive scores correlated negatively with MCS for the PFC/LLTL pair (*r*_19_ = −0.54, *p* = 0.017) and the PFC/PCC pair (*r*_20_ = −0.46, *p* = 0.041). In addition, PANSS Activation scores correlated negatively with MCS for the RLTL/RIPL pair (*r*_21_ = −0.69, *p* < 0.001, *p_FDR_* = 0.008), and this relationship survived FDR correction. Note that the variables that comprise the RLTL/RIPL factor load negatively with respect to each other, so a negative correlation means that higher levels of cross-modal connectivity are reflective of higher PANSS Activation scores.

## 4. Discussion

In this study, we examined the relationship between resting state functional connectivity and structural connectivity in the DMN, in patients with SZ and healthy controls, using the same ROI pairs in the same anatomical space. In general, data from the two modalities were not correlated, but those that were correlated involved connections of the PFC, PCC, and lateral temporal lobes with frontal and parietal regions. This is consistent with well-known frontotemporoparietal network dysfunction in SZ [65]. Moreover, MCS correlated with psychiatric symptoms in patients, suggesting that this approach has clinical significance in SZ. In addition to the methodological advantages of this multimodal approach, the results have implications for neuropathology and clinical symptomatology.

In particular, the results suggest that there is no imperative link between structural and functional connectivity for a given region pair within the DMN. This is consistent with several previous studies, showing that the correspondence between structural and functional connectivity profiles varies across neural networks. Specifically, in healthy adult samples, strong coupling is observed in primary sensory areas, while there is significant divergence between structure and function in the DMN [66,67]. Thus, low frequency oscillations, between disparate region pairs that comprise networks, can be synchronized by a third region, or that synchrony may dynamically vary across the scan, such that their relationship to structural connections may only arise in specific windows of time. Moreover, structure/function relationships in the DMN may be influenced by other networks that show significant alterations in schizophrenia (e.g., the SN and CEN [22,68]). Conversely, regions may be structurally connected but show little RSFC. In these latter cases, it may be that they are synchronized at higher frequency ranges that cannot be interrogated by BOLD fMRI. Future research with EEG/MEG would be helpful in terms of examining these issues. In addition, arterial spin labeling (ASL perfusion) would be another interesting avenue to pursue to provide information about regional cerebral blood flow. 

Fornito and Bullmore [38] have offered other explanations for findings of higher functional connectivity in SZ in the context of reduced DTI parameters. One of these involves the fact that DTI is a relatively imperfect measure of structural connectivity. In particular, the influence of crossing fibers in neurodevelopmental disorders, such as SZ, had been understudied. Another is that the later-developing association cortical hubs become miswired, as part of the SZ disease process, with higher functional connectivity being either a compensatory or a pathological response to such miswiring.

DMN RSFC did not differ between groups in this study. As noted in the Introduction, most studies show increased RSFC(Z) in the DMN in SZ, but some studies do not show differences, and group differences may vary across different degrees of clinical outcome. This could be due to heterogeneity in the sample. Alternatively, it is possible that the results may depend on the specific correlation measure used to estimate RSFC(Z).

In some cases, correlations between RSFC(Z) and NS were found for several region pairs in patients but not controls. Importantly, these correlations survive FDR correction and control for age, medication dosages, and ICV. In some ways, this is surprising, especially because across region pairs, NS was significantly higher in controls than patients. It may be that, in patients, lower levels of RSFC require structural supports for these region pairs, whereas in controls, RSFC can be maintained by more flexible interactions among brain systems. This aligns with prior work showing low structural-functional convergence in the DMN among healthy adults [66,67], as well as a recent study demonstrating increased structural-functional coupling of long-distance connections among offspring of parents with SZ, relative to healthy controls [34]. It is also consistent with notions of reduced complexity, in the brains of patients with SZ [69,70], as well as reduced flexibility among patients relative to controls. A recent study showing reduced temporal dynamics of DMN RSFC in SZ [71] is consistent with this idea, in that it would provide a clearer picture of whether summary measures across an entire scan are representative of how connectivity changes occur throughout that scan. 

Conjoint structural and functional connectivity was quantified by calculating MCS in each of the pairs that showed significant correlations between imaging parameters, and associations with clinical symptoms (PANSS) were assessed in correlational analyses. Here, we found that the Positive subscale correlated negatively with RSFC-NS MCS for the PFC/LLTL and PFC/PCC pairs. These correlations did not survive FDR correction. Higher scores on the Activation subscale were associated with higher connectivity of the RSFC-NS MCS for the RLTL/RIPL pair, which survived FDR correction. The negative correlation with the RSFC-NS RLTL/RIPL pair is, paradoxically, a positive relationship because the component scores within it are negatively correlated with each other. 

These relationships might be explained through a consideration of the phenomenology of each of the PANSS subscales. The Positive subscale is comprised of the Delusions, Unusual thought, Grandiosity, and Hallucinations items. The Activation subscale is comprised of the Hostility, Impulsivity, Excitement, and Uncooperativeness PANSS items. We speculate that the (net) positive correlation of RLTL/RIPL RSFC-NS MCS with the Activation subscale may indicate that higher multimodal connectivity is associated with outward-directed (externalizing) behavior, whereas the negative correlations for the positive symptoms scale may relate to psychosis per se. Further work will be necessary to determine whether this is, in fact, the case.

It is striking that diffusion parameters of FA and NS were, in general, poorly correlated (data not shown). Thus, a larger number of fibers does not necessarily correlate with higher FA. This supports the idea that those metrics provide unique information about diffusion properties. It must be borne in mind, however, that the tracts examined here are part of the DMN, differences are seen throughout the brain in other regions in SZ, and we are aware of no articles examining the specific tracts of the DMN in SZ. 

We examined correlations among structural connectivity, quantified using FA and number of streamlines (NS), as well as RSFC(Z) for region pairs within the DMN. These pairs were of two kinds: homologous (for directly corresponding pairs of regions, e.g., tracts and RSFC(Z) between the PFC and PCC nodes) and nonhomologous (for regions that did not directly correspond, e.g., tracts between PFC and PCC nodes that might significantly correlate with RSFC between PFC and RLOFC nodes). In this paper, we focused on homologous connections, but we present the nonhomologous connections in Table 2 and Table 3. We suggest that the latter provide indirect structural pathways that might be used to support functional networks, and they highlight that the relationship between structural and functional connectivity is likely to be complex.

Several other nonstructural mechanisms likely support functional networks. Cerebrovascular/neuronal coupling is known to play an important role in the BOLD signal [72,73]. It is likely that a considerable amount of variance in fMRI based RSFC may be explained by this mechanism, and the fact that the signal itself is obtained in a BOLD contrast makes it somewhat confounded. Basic neuroscientific studies are likely to be needed to examine this relationship.

Several important caveats should be borne in mind. First, DTI is an indirect measure of white matter organization, as it measures hindered diffusion in the brain, which tends to follow white matter tracts. Second, tractography is somewhat imprecise and is essentially an estimate of the primary pathway of hindered diffusion across voxels. The axons that are the source of such hindrance is on the order of microns, whereas the resolution of even the highest resolution source data is at the millimeter level. DTI findings could be verified in postmortem studies of white matter. Moreover, the number of streamlines does not necessarily bear a close relationship to the actual number of tracts in a white matter bundle. Third, resting state functional connectivity is a measure of low frequency synchrony (e.g., 0.01–0.1 Hz) of the BOLD signal across disparate regions. It will be important to examine other frequency bands in future work. The BOLD signal is sluggish, so it is likely that EEG studies will be helpful going forward. In addition, the BOLD signal itself is an indirect measure of neural activity, as noted above. 

There were some limitations to this study. First, the sample size was relatively small in this preliminary study, which limited statistical power. This article is primarily designed to show the utility of the FATCAT method in SZ. For this reason, although we adjusted results for false discovery rate, we also present uncorrected results. It is hoped that the uncorrected results will be replicated in larger samples. Second, the imaging sequences were relatively short single-band acquisitions. Third, higher numbers of diffusion directions and multishell diffusion acquisitions are likely to provide better estimates of diffusion, providing richer and more stable tractographic data. The relative brevity of our RSFC sequence restricted our ability to examine dynamic changes in RSFC that might have revealed brain states better related to tractographic measures than our static measures, and longer scans have greater reliability than a 6-min scan. This will be an important topic for future research. Finally, patients were chronically ill and on long-term regimens of antipsychotic medication. It would be important to study medication-naïve first episode participants.

## 5. Conclusions

In conclusion, we developed a method to directly integrate DTI and RSFC data using network-specific ROIs and scans from the same session. In many cases, data from the two modalities were not correlated, and when they were, they were not necessarily correlated in the expected pattern. We also showed that these relationships had clinical significance in patients. We suggest that it will be useful to have such an analytic framework to parse how connectivity is mediated and how it varies across clinical populations.

## Figures and Tables

**Table 1:** Demographic data for the sample.

	Patients		Controls			
Variable	*M*	*SD*	*M*	*SD*	*t*/χ2	*p*
Age (years)	38.8	10.5	38.2	8.0	−0.22	0.83
Sex (M/F)	23/6		17/8		0.89	0.34
ICV (cc) ^1^	1583.6	182.6	1547.4	151.1	−0.79	0.43
PANSS						
Positive	12.2	3.8	--	--		
Negative	16.1	5.3	--	--		
Activation	9.4	4.5	--	--		
Dysphoric	11.8	4.3				
Autistic	12.4	2.7				
Total	76.3	10.8	--	--		
Illness duration (years) ^2^	17.87	8.07	--	--		
CPZ equiv	1196.7	800.8	--	--		

Note: ^1^ Intracranial volume (from Freesurfer), ^2^ available for 14 patients, PANSS = Positive and Negative Syndrome Scale, CPZ equiv = chlorpromazine equivalents.

**Table 2:** Pearson correlations (r) between resting state functional connectivity and fractional anisotropy for region pairs.

**Controls**										
**Resting State Functional Connectivity**										
**Fractional Anisotropy**	**N**	**PFC-LLTL**	**PFC-PCC**	**PFC-RLTL**	**PFC-RLOFC**	**LLTL-PCC**	**PCC-LPHP**	**PCC-RPHP**	**RLTL-RIPL**	**RLTL-RLOFC**
PFC-LLTL	23	0.085	0.132	−0.155	−0.038	0.431 *	0.435 *	−0.097	0.37	0.081
PFC-PCC	23	−0.225	−0.254	−0.373	−0.291	−0.025	0.420 *	−0.07	−0.002	0.101
PFC-RLTL	21	−0.291	0.009	−0.23	−0.005	−0.05	0.518 *	0.34	−0.105	0.038
PFC-RLOFC	22	0.079	0.347	0.021	0.168	0.406	0.399	0.107	0.394	0.249
LLTL-PCC	20	0.055	−0.185	−0.042	0.125	0.136	0.219	0.044	0.405	0.243
PCC-LPHP	23	−0.115	0.016	−0.115	0.053	−0.026	0.455 *	−0.009	0.051	0.312
PCC-RPHP	22	−0.153	−0.064	−0.149	−0.051	−0.057	0.174	−0.291	0.334	0.403
RLTL-RIPL	23	0.028	0.024	0.007	−0.077	0.223	0.157	0	0.22	0.332
RLTL-RLOFC	23	−0.017	−0.131	−0.13	0.21	0.096	0.038	0.003	0.125	0.291
**Patients**										
**Resting State Functional Connectivity**										
**Fractional Anisotropy**	**N**	**PFC-LLTL**	**PFC-PCC**	**PFC-RLTL**	**PFC-RLOFC**	**LLTL-PCC**	**PCC-LPHP**	**PCC-RPHP**	**RLTL-RIPL**	**RLTL-RLOFC**
PFC-LLTL	27	−0.267	−0.341	−0.077	0.184	−0.564 **	−0.032	0.014	−0.277	0.100
PFC-PCC	27	0.088	0.005	−0.202	−0.093	0.044	0.454 *	0.098	−0.367	−0.199
PFC-RLTL	23	0.203	0.09	0.259	−0.004	−0.196	0.108	0.492 *	−0.251	0.192
PFC-RLOFC	27	−0.121	−0.238	−0.18	0.161	−0.354	0.192	0.115	−0.649 **	−0.043
LLTL-PCC	27	−0.145	−0.042	0.26	0.054	−0.153	0.024	0.314	−0.261	0.213
PCC-LPHP	29	−0.059	−0.391 *	−0.26	−0.123	−0.294	0.064	0.278	−0.514 **	−0.220
PCC-RPHP	27	−0.119	−0.344	0.07	−0.034	−0.164	0.294	0.096	−0.056	−0.175
RLTL-RIPL	29	0.171	−0.185	−0.066	0.269	−0.214	0.007	−0.02	−0.384 *	0.049
RLTL-RLOFC	28	0.451 *	0.29	0.27	0.163	0.05	0.163	0.132	0.01	0.145

Note. ** Pearson Correlation is significant at the 0.01 level (2-tailed), * Correlation is significant at the 0.05 level (2-tailed). Abbreviations: PFC = Prefrontal cortex; LLTL = left lateral temporal lobe; PCC = posterior cingulate cortex; RLTL = right lateral temporal lobe; RLOFC = right lateral orbitofrontal cortex; RIPL = right inferior parietal lobule.

**Table 3:** Spearman correlations (*ρ*) between resting state functional connectivity and number of streamlines for region pairs.

**Controls**										
**Resting State Functional Connectivity**										
**Number of Streamlines**	**N**	**PFC-LLTL**	**PFC-PCC**	**PFC-RLTL**	**PFC-RLOFC**	**LLTL-PCC**	**PCC-LPHP**	**PCC-RPHP**	**RLTL-RIPL**	**RLTL-RLOFC**
PFC-LLTL	23	−0.474 *	−0.121	−0.277	−0.018	−0.245	−0.305	−0.125	0.011	−0.335
PFC-PCC	23	−0.161	−0.280	−0.349	−0.244	−0.302	0.111	−0.107	−0.115	−0.203
PFC-RLTL	21	−0.049	−0.364	0.132	0.225	−0.184	−0.466 *	−0.096	0.327	0.253
PFC-RLOFC	22	−0.147	**−0.197**	0.057	−0.010	−0.359	−0.234	−0.080	−0.134	0.411
LLTL-PCC	20	0.367	0.136	0.592 *	0.320	0.045	0.024	0.305	−0.008	0.238
PCC-LPHP	23	0.111	−0.007	0.125	0.108	0.207	0.022	0.292	−0.076	−0.290
PCC-RPHP	22	−0.182	−0.284	−0.116	−0.023	−0.090	0.307	0.232	−0.058	0.063
RLTL-RIPL	23	−0.200	−0.209	−0.208	−0.148	−0.207	−0.264	−0.316	−0.170	0.103
RLTL-RLOFC	23	−0.179	−0.044	−0.232	−0.208	0.028	0.161	0.108	−−0.040	−0.147
**Patients**										
**Resting State Functional Connectivity**										
**Number of Streamlines**	**N**	**PFC-LLTL**	**PFC-PCC**	**PFC-RLTL**	**PFC-RLOFC**	**LLTL-PCC**	**PCC-LPHP**	**PCC-RPHP**	**RLTL-RIPL**	**RLTL-RLOFC**
PFC-LLTL	27	0.330	0.505 **	0.253	0.507 **	0.198	0.075	0.078	−0.032	0.341
PFC-PCC	27	0.386 *	0.524 **	0.081	0.245	0.374	−0.153	−0.125	−0.434 *	0.383 *
PFC-RLTL	23	0.123	0.292	0.367	0.160	0.236	0.113	0.235	0.267	0.109
PFC-RLOFC	27	0.028	0.375	0.216	−0.105	0.416 *	0.122	−0.150	0.096	0.085
LLTL-PCC	27	0.278	−0.053	0.128	−0.031	−0.171	0.039	0.104	0.011	−0.005
PCC-LPHP	29	0.220	0.064	0.194	0.162	−0.067	−0.041	0.094	0.160	−0.047
PCC-RPHP	27	0.154	0.183	0.120	0.353	0.045	0.048	−0.021	0.269	0.013
RLTL-RIPL	29	−0.039	−0.006	−0.278	−0.078	−0.128	−0.244	0.156	**−0.501** **	−0.086
RLTL-RLOFC	29	0.244	0.253	0.038	0.042	0.269	−0.123	0.109	0.207	0.114

Note. ** Correlation is significant at the 0.01 level (2-tailed), * Correlation is significant at the 0.05 level (2-tailed). BOLD = significant by Holm-Sidak FDR procedure. Values above are Spearman’s *ρ*. Abbreviations: PFC = Prefrontal cortex; LLTL = left lateral temporal lobe; PCC = posterior cingulate cortex; RLTL = right lateral temporal lobe; RLOFC = right lateral orbitofrontal cortex; RIPL = right inferior parietal lobule.

## Data Availability

The de-identified data that support the findings of this study are available from the corresponding author upon reasonable request.

## Supplementary Materials

The following are available online at https://www.mdpi.com/article/10.3390/brainsci12020156/s1, Table S1: Mean number of streamlines (NS) for each region pair, Table S2: Mean fractional anisotropy (FA) for each region pair, Table S3: Mean resting state functional connectivity (RSFC) for each region pair, Table S4. Partial correlations between RSFC(Z) and fractional anisotropy (FA) in patients controlling for age, medication dosage, and intracranial volume, Table S5. Spearman partial correlations between RSFC(Z) and fractional anisotropy (FA) in patients controlling for age, medication dosage, and intracranial volume. Table S6: Factor loadings for patients for multimodal connectivity scores for both Fractional Anisotropy (FA) and Number of Streamlines (NS).
