# Supplementary material for: Relationships between Diffusion Tensor Imaging and Resting State Functional Connectivity in Patients with Schizophrenia and Healthy Controls: A Preliminary Study
Source: Brain Sci. 2022 Jan 25;12(2):156. doi: 10.3390/brainsci12020156 (PMC8870342; doi:10.3390/brainsci12020156)
Supplement: Supplementary file 1 [file brainsci-12-00156-s001.zip › brainsci-1500462-supplementary.pdf]

Table S1. Mean number of streamlines (NS) for each region pair.

	Patients		Controls			
**Pair**	M	SD	M	SD	*U*	*p*
**PFC-LLTL**	5249.04	4950.38	7986.42	4095.53	181.0	.007
**PFC-PCC**	16617.96	8371.92	16690.25	6349.44	318.0	.910
**PFC-RLTL**	1191.13	1358.73	1401.86	1862.56	246.5	.883
**PFC-RLOFC**	3747.41	5288.74	3857.48	332.81	258.5	.311
**LLTL-PCC**	3700.41	4052.76	3245.67	5743.81	254.0	.540
**PCC-LPHP**	4599.59	3378.93	5729.50	3584.98	285.0	.260
**PCC-RPHP**	1973.74	1844.42	2815.96	2195.65	254.0	.271
**RLTL-RIPL**	7653.52	6281.45	9634.67	6382.67	272.0	.174
**RLTL-RLOFC**	3664.29	2813.64	3047.96	1988.19	304.0	.557

Note. M = mean; SD = standard deviation, U = Mann-Whitney *U* test-statistic. PFC = Prefrontal cortex; LLTL = left lateral temporal lobe; PCC = posterior cingulate cortex; RLTL = right lateral temporal lobe; RLOFC = right lateral orbitofrontal cortex; RIPL = right inferior parietal lobule

Table S2. Mean fractional anisotropy (FA) for each region pair.

	**Patients**		**Controls**				
**Pair**	M	SD	M	SD	*t*	*df*	*p*
	Fractional Anisotropy						
**PFC-LLTL**	.423	.021	.430	.017	1.185	49	.242
**PFC-PCC**	.417	.019	.424	.024	1.126	49	.266
**PFC-RLTL**	.426	.031	.433	.030	.786	43	.436
**PFC-RLOFC**	.413	.030	.428	.027	1.929	48	.060
**LLTL-PCC**	.435	.034	.443	.026	.821	46	.416
**PCC-LPHP**	.431	.027	.447	.027	2.176	51	.034
**PCC-RPHP**	.420	.033	.423	.033	.278	48	.783
**RLTL-RIPL**	.392	.019	.397	.020	1.074	51	.288
**RLTL-RLOFC**	.382	.034	.402	.028	2.383	50	.021

Note. M = mean; SD = standard deviation: PFC = Prefrontal cortex; LLTL = left lateral temporal lobe; PCC = posterior cingulate cortex; RLTL = right lateral temporal lobe; RLOFC = right lateral orbitofrontal cortex; RIPL = right inferior parietal lobule

Table S3. Mean resting state functional connectivity (RSFC(Z)) for each region pair.

	Patients		Controls				
**Pair**	M	SD	M	SD	*t*	*df*	*p*
				RSFC			
**PFC-LLTL**	1.174	.240	.992	.251	-2.689	51	.010
**PFC-PCC**	.898	.254	.883	.282	-.201	51	.841
**PFC-RLTL**	.817	.312	.721	.253	-1.208	51	.233
**PFC-RLOFC**	.713	.355	.861	.267	1.690	51	.097
**LLTL-PCC**	.863	.337	.726	.300	-1.539	51	.130
**PCC-LPHP**	.337	.140	.378	.220	.789	37.48[1]	.436
**PCC-RPHP**	.169	.219	.286	.286	1.682	51	.099
**RLTL-RIPL**	.751	.246	.819	.310	.901	51	.372
**RLTL-RLOFC**	.633	.317	.675	.207	.556	51	.580

Note. M = mean; SD = standard deviation: RSFC(Z) = r-to-z transformed resting state functional connectivity; PFC = Prefrontal cortex; LLTL = left lateral temporal lobe; PCC = posterior cingulate cortex; RLTL = right lateral temporal lobe; RLOFC = right lateral orbitofrontal cortex; RIPL = right inferior parietal lobule, [1]*t*-test for unequal variances.

Table S4. Partial correlations between RSFC(Z) and fractional anisotropy (FA) in patients controlling for age, medication dosage, and intracranial volume.

		Resting State Functional Connectivity								
**Fractional Anisotropy**	**dfs**	PFC-LLTL	PFC-PCC	PFC-RLTL	PFC-RLOFC	LLTL-PCC	PCC-LPHP	PCC-RPHP	RLTL-RIPL	RLTL-RLOFC
PFC-LLTL	21	-.185	-.106	-.014	-.129	.119	.282	-.047	.061	.160
PFC-PCC	21	-.280	-.239	-.300	-.101	-.218	.418*	.009	-.164	-.053
PFC-RLTL	17	-.189	.002	.059	-.189	-.022	.325	.420	-.157	.153
PFC-RLOFC	21	-.192	.032	-.018	-.054	.199	.360	.108	-.077	.172
LLTL-PCC	21	-.129	-.105	.169	-.084	.088	.133	.219	.041	.241
PCC-LPHP	23	-.320	-.294	-.252	-.308	-.036	.252	.152	-.178	-.016
PCC-RPHP	21	-.196	-.247	.028	-.145	-.050	.234	-.135	.189	.048
RLTL-RIPL	23	.104	-.048	.076	-.060	.170	.179	.055	-.135	.238
RLTL-RLOFC	22	.002	.012	.104	-.086	.192	.052	.117	.127	.228

Note. ** Pearson partial correlation is significant at the .01 level (2-tailed), * Pearson partial correlation is significant at the .05 level (2-tailed). Abbreviations: PFC = Prefrontal cortex; LLTL = left lateral temporal lobe; PCC = posterior cingulate cortex; RLTL = right lateral temporal lobe; RLOFC = right lateral orbitofrontal cortex; RIPL = right inferior parietal lobule.

Table S5. Spearman partial correlations between RSFC(Z) and fractional anisotropy (FA) in patients controlling for age, medication dosage, and intracranial volume.

		Resting State Functional Connectivity								
**Number of Streamlines**	**dfs**	PFC-LLTL	PFC-PCC	PFC-RLTL	PFC-RLOFC	LLTL-PCC	PCC-LPHP	PCC-RPHP	RLTL-RIPL	RLTL-RLOFC
PFC-LLTL	21	.175	.471	.333	.499*	.152	-.028	-.214	.060	.472*
PFC-PCC	21	.346	.506**	.128	.191	.384	-.242	-.312	.573**	.429*
PFC-RLTL	17	.091	.361	.365	.199	.243	-.183	-.173	.249	.196
PFC-RLOFC	21	-.023	.338	.158	.027	.375	.040	-.217	.058	.020
LLTL-PCC	21	.093	-.337	.073	-.051	-.389	-.175	.012	.023	-.053
PCC-LPHP	23	.169	.021	.248	.113	-.087	-.070	.061	.232	-.011
PCC-RPHP	21	.048	.147	.202	.301	.040	.034	-.109	.405	.077
RLTL-RIPL	23	.052	.034	-.254	-.109	-.100	-.238	.198	-.496*	-.047
RLTL-RLOFC	22	.152	.174	.021	.023	.220	-.242	.076	.240	.126

Note. ** Spearman partial correlation is significant at the .01 level (2-tailed), * Spearman partial correlation is significant at the .05 level (2-tailed). Abbreviations: PFC = Prefrontal cortex; LLTL = left lateral temporal lobe; PCC = posterior cingulate cortex; RLTL = right lateral temporal lobe; RLOFC = right lateral orbitofrontal cortex; RIPL = right inferior parietal lobule.

Table S6. Factor loadings for patients for multimodal connectivity scores for both Fractional Anisotropy (FA) and Number of Streamlines (NS).

**Fractional Anisotropy (FA)**				
**Measure**	Loading	Factor Eigenvalue	Bartlett's Test	$p_{Bartlett}$
**PFC_LLTL**	-.79 (FA)	1.27	1.81	.18
**PFC_PCC**	.71	1.00	.00	.98
**PFC_RLTL**	.79	1.26	1.42	.23
**PFC_RLOFC**	.76	1.16	.64	.42
**LLTL_PCC**	-.76 (FC)	1.15	.58	.45
**PCC_LPHP**	.73	1.06	.11	.74
**PCC_RPHP**	.74	1.10	.23	.63
**RLTL_RIPL**	-.83 (FC)	1.38	4.23	.04
**RLTL_RLOFC**	.76	1.14	.54	.46
**Number of Streamlines (NS)**				
**PFC_LLTL**	.80	1.28	1.93	.16
**PFC_PCC**	.86	1.48	6.30	.012
**PFC_RLTL**	.86	1.48	5.29	.021
**PFC_RLOFC**	.81	1.30	2.30	.13
**LLTL_PCC**	-.72 (NS)	1.02	.013	.91
**PCC_LPHP**	-.71 (NS)	1.01	.002	.96
**PCC_RPHP**	.74	1.08	.17	.68
**RLTL_RIPL**	-.84 (NS)	1.42	5.23	.023
**RLTL_RLOFC**	-.73 (NS)	1.07	.12	.73

Note. Abbreviations: FA = fractional anisotropy, NS = number of streamlines; PFC = Prefrontal cortex; LLTL = left lateral temporal lobe; PCC = posterior cingulate cortex; RLTL = right lateral temporal lobe; RLOFC = right lateral orbitofrontal cortex; RIPL = right inferior parietal lobule. Under loading, the label in parentheses refers to which variable loads negatively on the eigenvariate.
